# Respiratory syncytial virus reduces STAT3 phosphorylation in human memory CD8 T cells stimulated with IL-21

**DOI:** 10.1038/s41598-019-54240-9

**Published:** 2019-11-28

**Authors:** Krist Helen Antunes, André Becker, Caroline Franceschina, Deise do Nascimento de Freitas, Isadora Lape, Mariana D’Ávila da Cunha, Lidiane Leitão, Mauricio M. Rigo, Leonardo Araújo Pinto, Renato T. Stein, Ana Paula Duarte de Souza

**Affiliations:** 1 Laboratory of Clinical and experimental Immunology, Infant Center, School of Medicine, Pontificia Universidade Católica do Rio Grande do Sul (PUCRS), Porto Alegre, Brazil; 2 Laboratory of Respiratory Physiology, Infant Center, School of Medicine, PUCRS, Porto Alegre, Brazil; 3 School of Health and Life Sciences, PUCRS, Porto Alegre, Brazil

**Keywords:** Immunological memory, Virus-host interactions, Respiratory tract diseases

## Abstract

Respiratory syncytial virus (RSV) is a common cause of childhood lower respiratory tract infections. The recent failure of a vaccine candidate based on recombinant F protein underlines the urgent need to better understand the protective human memory immune response against RSV. Signal transducer and activator of transcription 3 (STAT3) protein is a transcription factor that promotes the maturation of the memory CD8 T cell response in cooperation with IL-10 and IL-21. However, the role of STAT3 in the memory CD8 T cell response during RSV infection remains to be elucidated. We found that in infants with bronchiolitis infected with RSV, the expression of STAT3 detected in nasal washes is reduced when compared to that in infants infected by other viruses. *In vitro*, RSV impairs STAT3 phosphorylation induced by IL-21 in purified human memory CD8 T cells. In addition, RSV decreases granzyme B production by memory CD8 T cells, reducing its cytotoxic activity against RSV-infected epithelial pulmonary cell lines. Together, these data indicate that RSV modulates the IL-21/STAT3 pathway in human memory CD8 T cells, and this could be a mechanism to be further explored to improve the memory response against the infection.

## Introduction

Respiratory syncytial virus (RSV) is the leading pathogen of lower respiratory diseases in children less than 5 years old and is associated with bronchiolitis and pneumonia diagnoses. Globally, it is estimated that 59,000 children die per year from RSV infection^1^. RSV also causes severe respiratory disease in the elderly^2^ and in immunocompromised adult patients^3^. A prophylactic monoclonal antibody has been used to prevent disease in high-risk groups, but this drug is too costly and is impractical for universal use. Currently, there is no licensed vaccine available, and recent RSV vaccine candidate trial failures highlight gaps in knowledge regarding human immunological protection^4^, although maternal immunization shows promising results. Neutralizing antibodies and cellular immune responses are involved in protection against the virus, but these mechanisms are not yet fully understood^5^. Most importantly, natural infection by RSV in children does not establish long-lasting immunological memory^6,7^; consequently, individuals remain susceptible to repeated RSV infections throughout life.

Peripheral blood RSV-specific cell-mediated cytotoxic immune responses are more frequent in infants with bronchiolitis with mild infection than in those with severe infection^8^. In experimental RSV infection of adults, CD8 T cells are associated with a reduction in pulmonary viral load^9^. One proposed mechanism is that during RSV infection, there is suboptimal signaling to T cells, and therefore, a failure occurs in the generation of long-lived polyfunctional T cells^10^. A better understanding of the modulation of the CD8 T cell-mediated response by RSV is necessary to comprehend the protection and development of new vaccine approaches.

The signal transducer and activator of transcription 3 (STAT3) protein is a major transcription factor involved in many cellular processes, and its complete absence is lethal in mice^11^. STAT3 is key to modulating both innate and adaptive responses through several cytokine pathways. After cytokines bind to their receptors, a signaling pathway involving Janus kinase (JAK) induces STAT3 phosphorylation with consequent signaling to the nucleus^12^. In humans, a dominant-negative loss-of-function mutation in STAT3 causes autosomal hyper IgE syndrome (HIES)^13,14^. Patients with STAT3 mutations present with more viral infections^15,16^ and show impairment in memory CD8 T cell responses^17^. Accordingly, in mice, STAT3 promotes and preserves the memory cell potential in virus-specific CD8 T cells, sustaining the expression of transcription factors essential for the memory response^18^. The role of STAT3 in regulating the CD8 memory response is associated with IL-10 and IL-21. However, the roles of IL-21 and STAT3 in the memory CD8 T cell response during RSV infection are unknown, and addressing this issue is the main goal of the present study.

## Results

### RSV infection reduces STAT3 expression in nasal washes from infants with bronchiolitis

**Figure 1:**
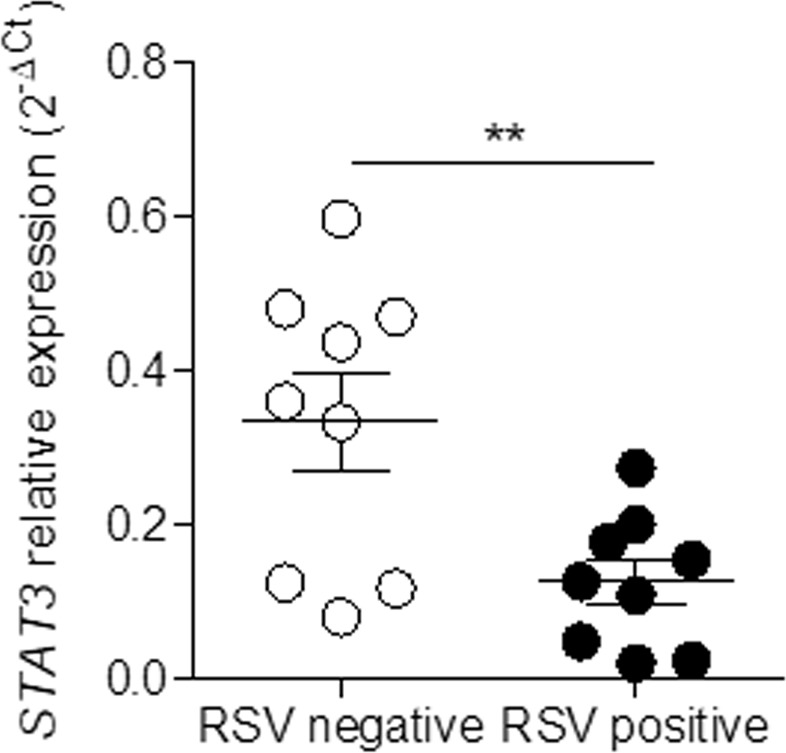
The expression of the *STAT3* gene is reduced in children with bronchiolitis positive for RSV. Nasal wash samples were collected from infants up to 12 months of age who were admitted to the hospital with acute bronchiolitis. The presence of RSV was confirmed by real-time PCR. STAT3 gene expression was assessed by real-time PCR and compared with endogenous gene expression (unpaired *t* test, **p = 0.0078).

To assess the importance of STAT3 during the time of acute RSV infection, we used real-time PCR to analyze STAT3 expression in nasal samples from children infected with RSV. There were no significant differences in age, gender distribution or birth weight among patients from the RSV-positive group compared to the RSV-negative group (Table 1). Samples from infants who were positive for RSV (n = 9) had reduced expression of the *STAT3* gene (mean = 0.1269 and SD = 0.08546) compared to samples from children who were not positive for RSV (n = 9) (mean = 0.3337 and SD = 0.1852), p = 0.0078 (Fig. 1).

**Table 1:** Patient characteristics.

	RSV positive (n = 9)	RSV negative (n = 9)	*P* value
Age (months)	3.100 ± 1.892	4.490 ± 3.089	0.2239
Sex (M/F)	7/2	6/3	0.9185*
Birth weight (kg)	3.096 ± 0.5362	3.188 ± 0.4547	0.6793

Values are presented as the mean ± standard deviation for age and birth weight (unpaired *t* test analysis).

*P value for chi-square test.

RSV = respiratory syncytial virus; M = male; F = female.

### RSV impairs STAT3 phosphorylation induced by IL-21 in purified human memory CD8 T cells

**Figure 2:**
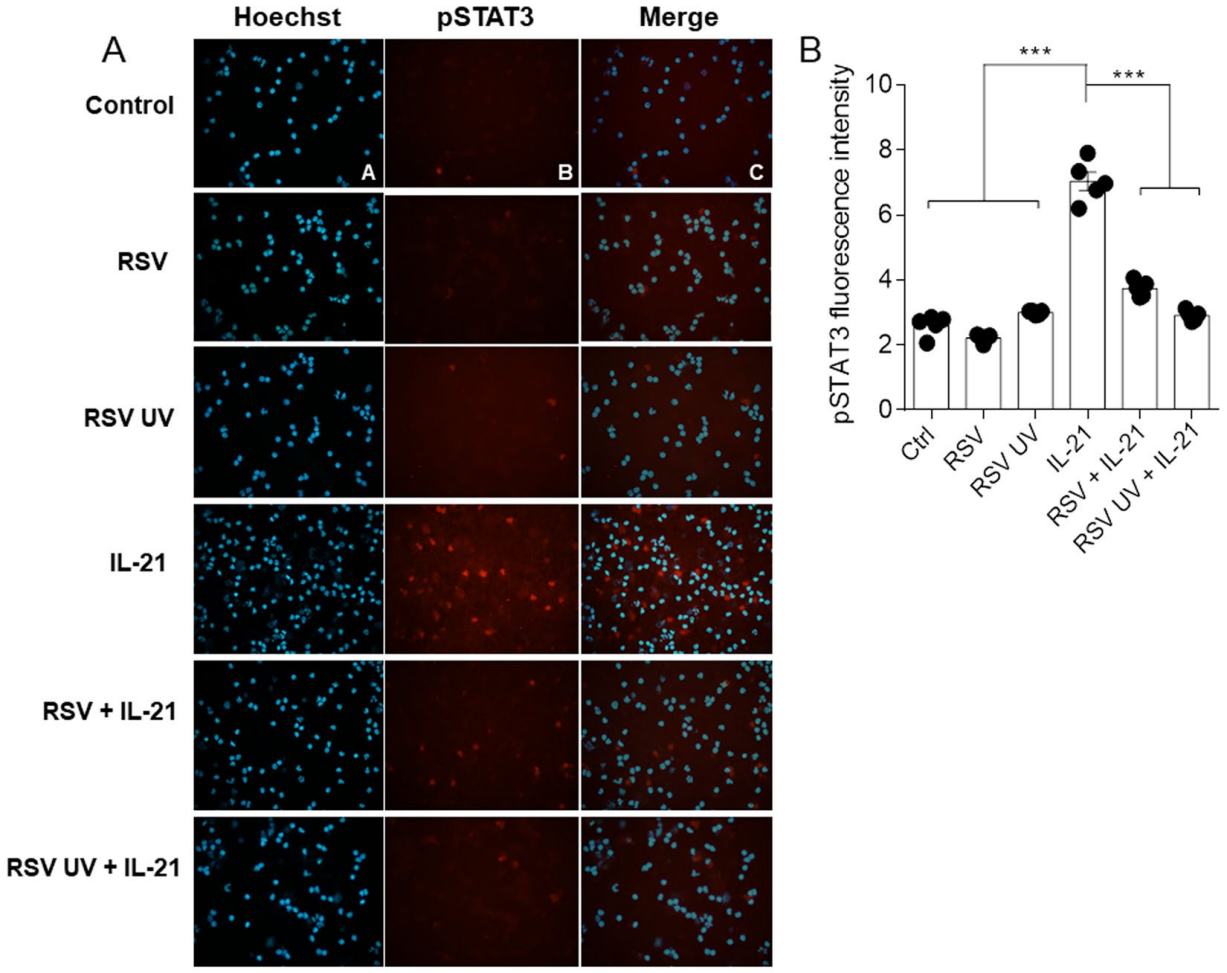
RSV inhibits pSTAT3 induced by IL-21 in purified human memory CD8 T cells. Human memory CD8 T cells were isolated from PBMCs, incubated with RSV (5 × 10² PFU/ml) for 1 h and treated with IL-21 (25 ng/ml). After 30 min, the cells were harvested, fixed and stained for immunofluorescence analysis. (**A**) Fluorescence images of cell nuclei stained for Hoechst (blue) and pSTAT3 Ser727 (red). (**B**) Quantification of STAT3 phosphorylation on Ser727in purified human CD8 T cells.

**Figure 3:**
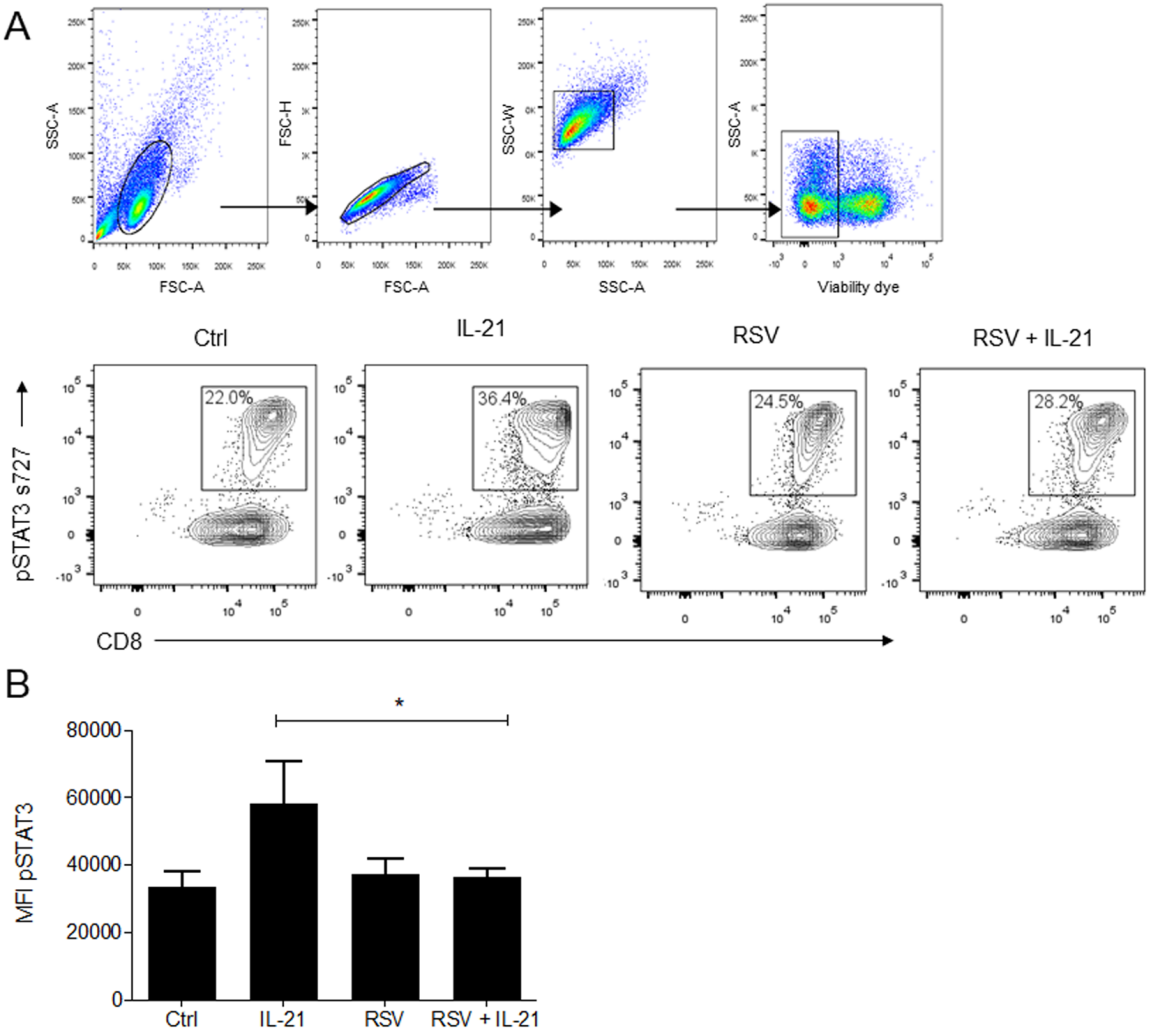
RSV inhibits pSTAT3 induced by IL-21 in live purified human memory CD8 T cells. Human memory CD8 T cells were isolated from PBMCs, incubated with RSV (5 × 10² PFU/ml) for 1 h and treated with IL-21 (25 ng/ml). After 30 min, the cells were harvested and stained for flow cytometry analysis. (**A**) Gate strategy and representative plots of flow cytometry analysis of pSTAT3 in memory CD8 T cells. (**B**) MFI (mean of fluorescence intensity) of pSTAT3 in memory CD8 T cells. Data are expressed as the mean ± SEM. Statistical significance was determined using one-way ANOVA followed by Tukey’s multiple comparison test. *p < 0.05, **p < 0.01, ***p < 0.001.

Given the importance of STAT3 signaling on human memory CD8 T cells, we further measured STAT3 phosphorylation on serine 727 in cells isolated from the blood of healthy adult subjects. Human CD8 +CD45RO +CD45RA-CD56-CD57- memory T cells were obtained from PBMCs by magnetic isolation. Purified memory CD8 T cells were incubated with RSV for 1 h, and pSTAT3 was measured by immunofluorescence. We found that RSV was not able to directly modulate STAT3 activation in purified memory CD8 T cells (Fig. 2). We hypothesized that this scenario might change after adding a T cell stimulus. Since IL-21 plays an important role in memory CD8 T cell responses by activating STAT3, purified human memory CD8 T cells were incubated with RSV for 1 h and subsequently stimulated with IL-21 for 30 min to measure pSTAT3. IL-21 treatment induced STAT3 phosphorylation (Fig. 2). Interestingly, RSV impaired the induction of STAT3 phosphorylation by IL-21 in human memory CD8 T cells (Fig. 2). UV-inactivated virus presented similar effects on the induction of pSTAT3 by IL-21 (Fig. 2). We confirmed that RSV was indeed impairing STAT3 phosphorylation mediated by IL-21 in living purified memory CD8 T cells using flow cytometry (Fig. 3).

*In silico* prediction shows that IL-21 and RSV G protein, but not RSV F protein, can interact with IL-21R.

Since these data indicated that RSV infectivity was not mandatory for the modulation of STAT3 phosphorylation in human memory CD8 T cells stimulated with IL-21 *in vitro*, we further evaluated if a protein present on the surface of the virus could be responsible for the inhibition of pSTAT3 mediated by IL-21. RSV Protein F was not able to inhibit the phosphorylation of STAT3 mediated by IL-21 in memory CD8 T cells *in vitro* (Supplementary Fig. 1). As protein F is described to interact with TLR4, we also analyzed if RSV could reduce STAT3 phosphorylation induced by LPS. Although there was a reduction in MFI of STAT3 when RSV was added before LPS stimulation on purified memory CD8 T cells, this effect was not significant different from LPS alone (Supplementary Fig. 2).

**Figure 4:**
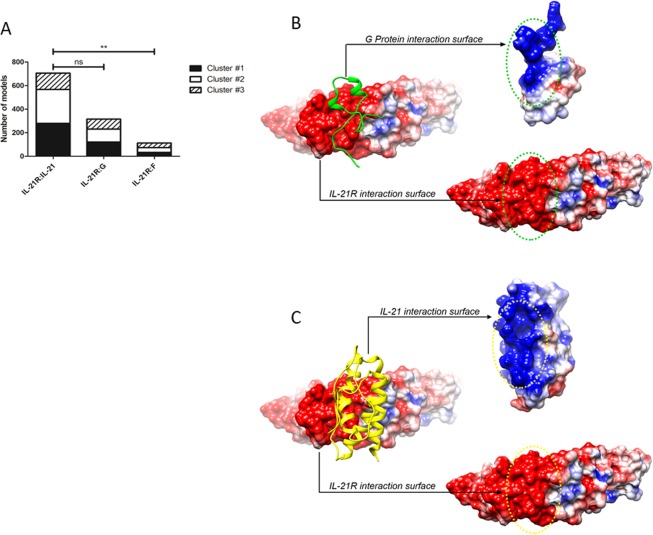
IL-21 and RSV G protein possess the same electrostatic potential pattern in the region involved in the interaction with IL-21R. (**A**) Number of interaction models (Clusters #1, #2, and #3) between the receptor (IL-21R) and the proteins analyzed (IL-21, RSV G protein, and RSV F protein). The main clusters of models were retrieved from ClusPro and compared. Statistical significance was determined using one-way ANOVA followed by Bonferroni’s Multiple Comparison Test. **p < 0.01 (**B,C**) Both proteins compete for the same binding spot. The charges distribution and surface calculation were performed with UCSF Chimera tools (electrostatic potential rages from -5 to + 5 kcal/(mol*e), where the colors blue, white, and red represent positive, neutral, or negative charges, respectively).

Based on our data that RSV impaired the induction of STAT3 phosphorylation mediated by IL-21, we speculate if RSV surface proteins could have a role in the interaction with IL-21 receptor (IL-21-R). We simulated the interaction of IL-21R with IL-21 (positive control), RSV G protein and RSV F protein. The algorithm was able to reproduce the interaction of IL-21R with IL-21, producing a large number of models. Several models were also generated for the interaction between IL-21R and RSV G protein. However, the same does not occur for the interaction between IL-21R and RSV F protein (Fig. 4A). These data suggest that IL-21R can interact with both, IL-21 and RSV G protein, but not with RSV F protein.

We next analyzed the representative models from the main interaction clusters to evaluate the binding mode of IL-21R with IL-21 and RSV G protein. Our data suggest that this interaction occurs mainly due to complementarity of charges and structure topography at a specific region in the interaction surface of the proteins. Interestingly, IL-21 and RSV G proteins share the same electrostatic pattern (positively charged residues) in the region involved in the interaction with IL-21R (negatively charged residues) (Fig. 4B,C). Moreover, both proteins were predicted to interact in the same binding spot highlighting a possible competition of IL-21 and RSV G protein for IL-21R (Supplementary Fig. 3).

### RSV diminishes granzyme B production in memory CD8 T cells, reducing its cytotoxic activity against RSV-infected epithelial pulmonary cell lines

**Figure 5:**
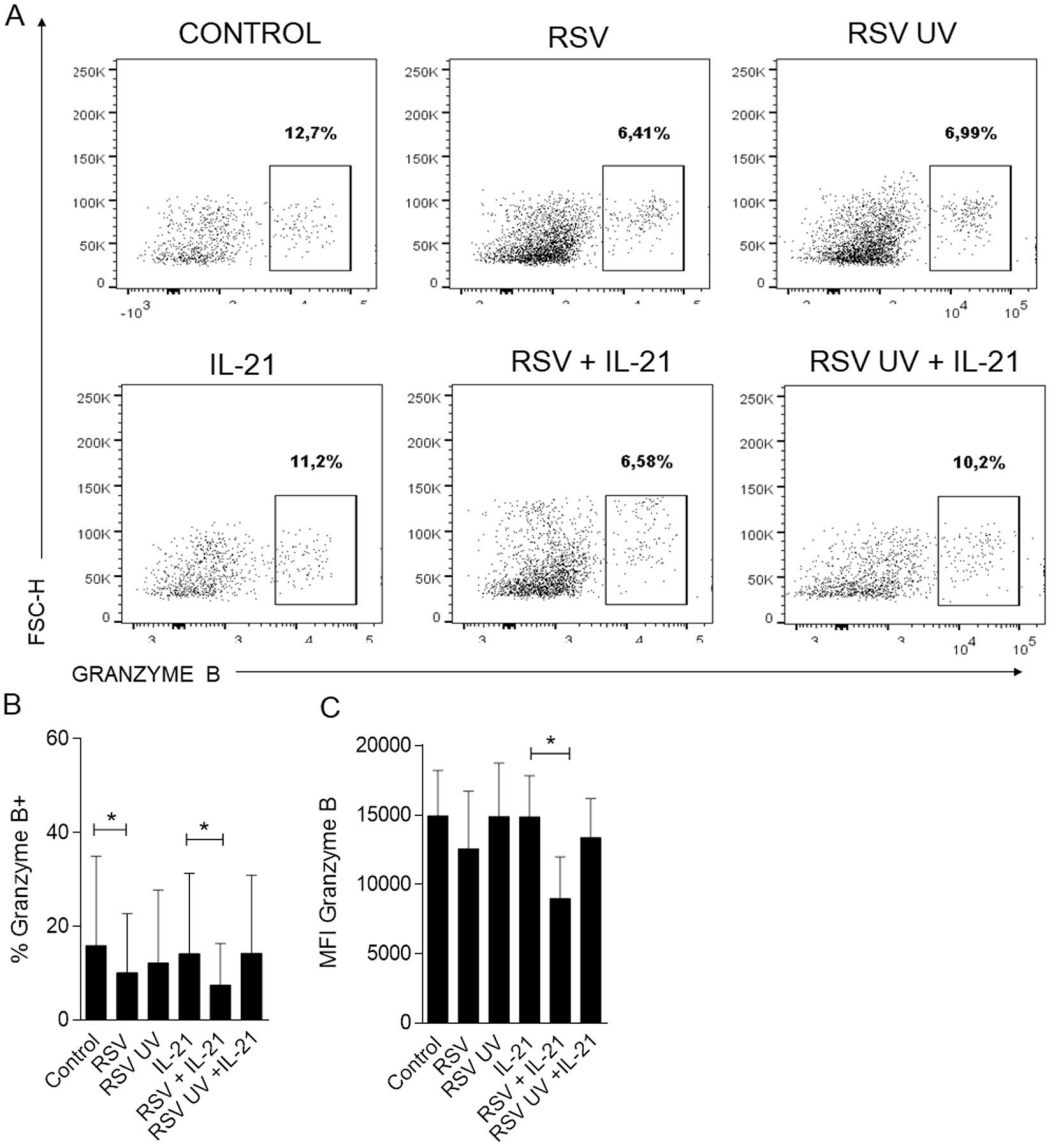
RSV reduces granzyme production in IL-21-treated human CD8 T cells. Human memory CD8 T cells were isolated from PBMCs, incubated with RSV for 1 h and stimulated with IL-21 for 4 h Granzyme B production was analyzed by flow cytometry. (**A**) Representative flow cytometry plots from memory CD8 T cells positive for granzyme B. (**B**) Percentage of memory CD8 granzyme B+ cells. (**C**) MFI of granzyme B+ cells. Statistical significance was determined using the Friedman test followed by the Dunn posttest.

To check if RSV could modulate memory CD8 T cell granzyme B production in response to IL-21, memory cells were incubated with RSV for 1 h and subsequently stimulated with IL-21 for 4 h. RSV infection decreased the production of granzyme B by memory CD8 T cells in the presence of IL-21 (Fig. 5). When the virus was UV inactivated, this effect was abolished (Fig. 5).

**Figure 6:**
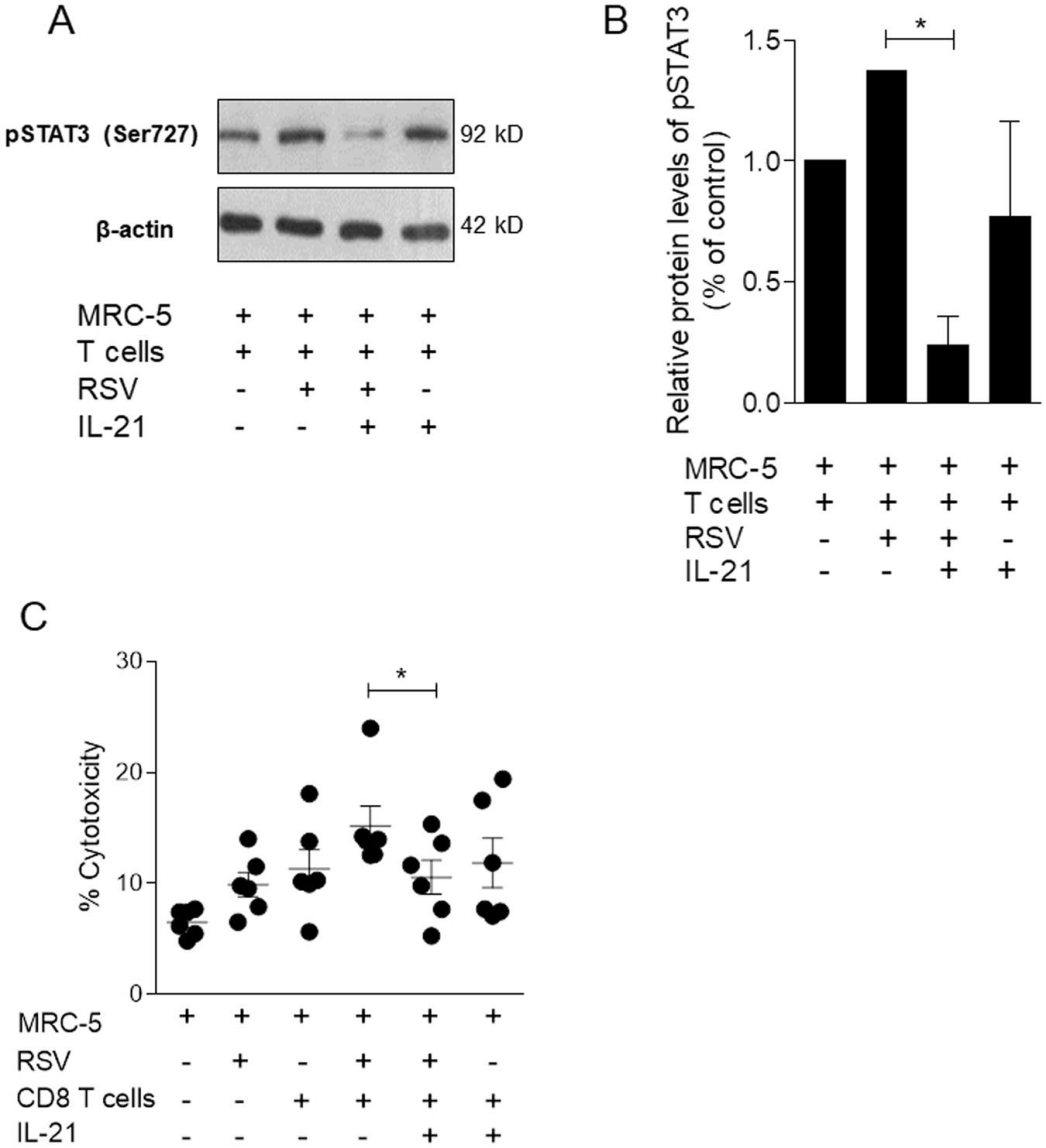
Pulmonary cells infected with RSV inhibit STAT3 phosphorylation and cytotoxic activity in IL-21-treated human CD8 T cells. Human pulmonary cells (MRC5) were infected with RSV (1 × 10^4^ PFU/ml) for 1 h. Afterwards, the medium was replenished, and human CD8 T cells isolated from PBMCs were placed in coculture with the infected MRC5 cells and then stimulated with IL-21 (25 ng/ml) for 30 min. (**A**) Cropped blot bands of total phosphorylated STAT3 protein (on serine 727) in memory CD8 T cells analyzed by Western blot. β-Actin was used to normalize protein quantification. (**B**) pSTAT3 protein quantification from Western blot analysis. Data are shown as percentages over untreated/uninfected controls. Full-length blots are shown in Supplementary Fig. 4. (**C**) Human pulmonary MRC-5 cells (1 × 10^5^ cells) were infected with RSV and cocultured in 96-well plates with human memory CD8 T cells (8 × 10^4^ cells) stimulated with IL-21 (25 ng/ml) in RPMI with 2% FBS. Human CD8 T cell cytotoxicity against MRC-5 cells was assessed by lactate dehydrogenase detection in the coculture supernatant. Data are expressed as the mean ± SEM. Statistical significance was determined using one-way ANOVA followed by Tukey’s multiple comparison test. *p < 0.05, **p < 0.01, ***p < 0.001.

We also measured pSTAT3 expression in memory CD8 T cells cocultured with RSV-infected pulmonary cells (MRC-5). The expression of pSTAT3 in IL-21-treated memory CD8 T cells was reduced compared to that in untreated cells when cocultured with RSV-infected pulmonary cells (Fig. 6A,B). Next, we evaluated the capacity of IL-21-stimulated memory CD8 T cells to kill RSV-infected MRC-5 cells after 12 h of culture and found that the cytotoxic effect of memory CD8 T cells is diminished in the presence of IL-21 (Fig. 6C).

## Discussion

Memory CD8 T cells are important for vaccine design, and different vaccination strategies to generate virus-specific memory CD8 T cells have demonstrated that these cells are key mediators for protection against secondary infection in mouse models^19^. Indeed, a deeper understanding of human memory CD8 T cells is necessary to develop better RSV vaccines. In the present study, we demonstrated that children infected with RSV presented reduced *stat3* gene expression in nasal wash samples. Also, RSV was able to modulate the function of human memory CD8 T cells stimulated with IL-21 *in vitro* and impair STAT3 phosphorylation. In silico analysis suggested that RSV G protein could interact with IL21-R. Our data demonstrated that RSV affects the IL-21-STAT3 pathway, and this could be a newly described evasion mechanism associated with poor memory CD8 T cell responses during acute infection.

IL-21 is a pleiotropic cytokine that not only operates via the JAK–STAT3 pathway but also activates the PI3K–AKT and MAPK signaling cascades, both of which are involved in the modulation of the CD8 T cell response^20^. IL-21 influences the expression of transcription factors important for the T cell response, including T-bet, Eomes, Bcl6, and Blimp1. The importance of this cytokine in regulating CD8 T cell responses is demonstrated by its critical role in sustaining antiviral CD8 T cells during LCMV infection in mouse models^21^. Several studies have demonstrated that IL-21 is also required for an optimal recall of memory CD8 T cells responses^22–24^. During RSV infection in mice, IL-21 seems to be key in the process of viral clearance and in regulation of CD4 T cell responses^25^.

Other studies have demonstrated the role of RSV in inducing STAT signaling. Kong *et al*. demonstrated that RSV activates STAT1 and STAT3 in the pulmonary A549 cell line and that this activation is necessary for the successful infection of epithelial cells^26^ (Kong, 2003). The authors also found that STAT3 phosphorylation in infected pulmonary cells is mediated by the IL-6 production induced by RSV^26^. In murine bone marrow-derived dendritic cells RSV was able to prevent IFN-β-mediated phosphorylation and subsequent nuclear translocation of STAT-1 and STAT-2^27^. However, RSV infection did not affect STAT3 phosphorylation induced by IL-10^27^. These data indicate that RSV modulation of STAT3 differs depending on the type of cells analyzed. Here, we demonstrate that RSV can reduce STAT3 activation in memory CD8 T cells stimulated by IL-21 and that this modulation is independent of the RSV infective ability. We wonder if the RSV F protein was responsible for this inhibition, but this was not the case.

We assess *in silico* the interaction between IL-21R and the main surface RSV glycoproteins: RSV F protein and RSV G protein. As expected, the algorithm did not find a suitable number of models that would represent an interaction between IL-21R and RSV F protein. However, it did find a large cluster of models representing IL-21R and RSV G protein interaction. The analysis of the representative models shows a complementarity of charges and topography on the interaction site between both proteins. Moreover, the same IL-21R site and the same physical-chemical properties are shared by RSV G protein and the natural ligand of IL-21R, the IL-21. These data support the hypothesis that RSV is inhibiting STAT3 phosphorylation mediated by IL-21 in a mechanism associated with G protein interacting with IL-21R. RSV G protein is a glycoprotein conserved in all strains of RSV and has a central conserved cysteine noose region a CX3C chemokine motif. This motif interacts with CX3CR1 that is present in different cells^28^ and it is important for the RSV infectivity on human pulmonary epithelial cells^29^. CX3C motif has been associated with impairment of T cell response during infection^30^. Interestingly, IL-21 is associated with increased expression of the chemokine receptor CX3CR1 on T cells^31^. Our data suggest a new role for RSV G protein in blocking the ligation of IL-21 on IL-21R on memory CD8 T cells.

We found that RSV partially inhibits STAT3 phosphorylation induced by LPS on memory CD8 T cells. LPS induced STAT3 triggering TLR4 to produce IL-6^32^ and in human T cells mediated SOCS3 and STAT3 signaling pathway^33^. Interesting, it has been described that soluble G protein inhibits TLR4^34^ and SOCS3 is increased in cells infected with RSV^35^. Consequently, we cannot exclude that RSV inhibited STAT3 phosphorylation on memory CD8 T cells induced by other stimuli rather than IL-21.

We also found that RSV can decrease the production of granzyme B in the presence of IL-21 and that this effect is dependent on the RSV infective ability; this effect was not detected when the UV inactivated virus was used. These data suggest that reducing the level of STAT3 may not be essential to reduce granzyme B production in response to IL-21 during RSV infection because the UV inactivated virus can impair STAT3 phosphorylation but not necessarily affect granzyme B production. In mice, it has been demonstrated that STAT3 activation decreases the expression of cytotoxic genes and granzyme B production^36^. Additionally, it has been shown that murine splenic STAT3 knockout memory CD8 T cells present aberrant expression of granzyme B following viral clearance^18^. In contrast, IL-21 did not induce the *in vitro* production of granzyme B by resting human CD8 T cells, similar to our findings. However, IL-21 in association with IL-15 induced pSTAT3 in human CD8 T cells, which is required for the expression of granzyme B^17^. These data showed that STAT3 in human and murine CD8 T cells functions in different pathways, highlighting the importance of studies in human cells.

One limitation of our study is that we analyzed the expression of the *STAT3* gene in the nasal washes of infants infected with RSV by real-time PCR but not the phosphorylation of STAT3 protein. However, it has been described that activated STAT3 translocates to the nucleus and induces the transcription of a large number of genes, including the *STAT3* gene itself. Consequently, the lower expression of *STAT3* might be associated with reduced phosphorylation of STAT3.

In summary, our study described the role of STAT3 in RSV infection, especially considering the memory CD8 T cell response. These data might be important for future vaccine strategies against clinically severe RSV respiratory illness.

## Methods

### Human samples

We used nasopharyngeal lavage samples obtained from infants hospitalized with bronchiolitis in the first year of life. These samples came from patients who had up to 72 h of clinical signs or symptoms of a lower respiratory tract infection (cough, wheezing and/or respiratory distress). All patients underwent data collection and nasopharyngeal wash for the identification of respiratory viruses on the first day of hospitalization. RSV presence was identified by both direct immunofluorescence and RT-PCR.

After collection, nasal wash samples were diluted in 1 mL of saline, homogenized and centrifuged at 1800 rpm for 5 minutes. The supernatant was discarded, and the cell pellet was suspended in 100 µL of RNA*later* stabilization solution (ThermoFisher Scientific, MA, USA) and frozen at −80 °C until further analysis.

Peripheral blood mononuclear cells (PBMCs) were isolated from healthy male and female adult donors aged 18–25 years old. Twenty milliliters of blood was collected from donors after signing an informed consent form.

### Ethics

For infant sample collection, the parents or legal guardians of all participants signed the informed consent form before sample collection. The study was approved by the Research Ethics Committee of PUCRS (protocol no. 09/04 678). For the healthy donor sample collection, the adult donor signed informed consent form before sample collection. The study was approved by the Research Ethics Committee of PUCRS (protocol no. 844.206). All procedures followed the standards established by the Declaration of Helsinki.

### Real-time PCR analysis

Nasopharyngeal lavage samples were suspended in 1 mL of TRIzol reagent (ThermoFisher Scientific, MA, USA), and the protocol for total RNA extraction was carried out according to the manufacturer’s instructions. RNA was reverse transcribed into cDNA using a Superscript III Kit (ThermoFisher Scientific, MA, USA) and fluorometric quantification was performed with Qubit (ThermoFisher Scientific, MA, USA). The quality of the cDNA was tested by amplification of the β-actin endogenous gene using specific primers and probes for TaqMan Assay (HuACTB; Applied Biosystems - ThermoFisher Scientific, MA, USA) and 2X TaqMan Master Mix. PCR conditions were chosen based on the TaqMan Master Mix protocol. STAT3 expression was performed using specific primers and probes for TaqMan Assay (Hs00374280_m1) using the β-actin (HuACTB) and glyceraldehyde-3-phosphate dehydrogenase (GAPDH) (Hs03929097_g1) genes as endogenous controls. Quantification of gene expression was carried out using StepOne (Applied Biosystems – ThermoFisher Scientific, MA, USA). A total of 4 ng of cDNA was used for each reaction. The target gene expression was determined using the 2^−ΔCt^ method. The delta value was calculated by subtracting the CT value for β-actin or GAPDH from the CT value for *STAT3* for each of the samples.

### Virus

The RSV A2 strain was kindly provided by Fernando Polack, Fundacion Infant, Argentina. The virus was propagated in monkey kidney-derived epithelial cells (Vero) (ATCCCCL-81). After 3 days of incubation, infected cells were harvested and centrifuged at 1500 rpm for 10 minutes. The pelleted cells were freeze-thawed 3 times to extract all new viral particles. Viral aliquots were stored at -80 °C until quantification and use. To quantify viral plaque-forming units (PFU), dilutions of the virus were incubated on Vero cells in 24-well plates (KASVI, PR, Brazil) for 2 h at 37 °C. Cells were then overlaid with 500 μl of 0.5% carboxymethylcellulose (Sigma Aldrich, Germany) in 1X DMEM F12 (Gibco) and allowed to incubate for 4 days at 37 °C. After 4 days of incubation, cells were fixed with methanol (Sigma-Aldrich) and then stained with 1:1000 goat anti-RSV antibody (AB1128 – Millipore, Germany) followed by 1:2000 rabbit anti-goat IgG (H + L)-HRP (31402 – ThermoFisher Scientific, MA, USA). Plate imaging was performed following enzymatic digestion of 4-Chloro-1-naphthol solution by HRP (Sigma Aldrich, Germany).

### Isolation of human memory CD8 T cells

A total of 1 × 10^7^ cells/ml isolated from PBMCs from healthy donors were used for the purification of human memory CD8 T cells by magnetic beads using a human CD8+ Memory T Cell Isolation Kit (Miltenyi Biotec, Germany) according to the manufacturer’s instructions. Cells were kept in RPMI with 2% fetal bovine serum (FBS) on ice until the experiment was performed.

### Immunofluorescence

Human memory CD8 T cells (5 × 10^4^ cells) were seeded in 96-well plates (KASVI) containing RPMI 2% FBS. RSV or ultraviolet (UV)-inactivated RSV (5 × 10² PFU/ml) were added and cultured with the cells for 1 h before treatment with IL-21 (25 ng/ml) (ImmunoTools). After 30 min, the cells were centrifuged and set on a microscopy slide. Briefly, slides were fixed with acetone for 30 seconds and incubated with Fc Block (supernatant of 2.4G2 cells with 5% heat-inactivated human serum) for an additional 20 minutes. Cells were stained with 1:300 of phospho-STAT3 (Ser727) polyclonal antibody (PA5-17876 – ThermoFisher Scientific) followed by 1:2000 of secondary goat anti-mouse IgG1 PE antibody. Antibody dilutions and washes were performed in 1X PBS. Nuclear staining was performed with Hoechst 33342 (ThermoFisher Scientific), and slides were mounted with glycerin for analysis under a fluorescence microscope (Olympus). Images were acquired using CellSens software (Olympus). Fluorescence intensity was measured using ImageJ.

### Flow cytometry

The isolated human memory CD8 T cells (1 × 10^5^ cells) were incubated with RSV (5 × 10² PFU/ml) for 1 h and subsequently stimulated with IL-21 (25 ng/ml) for 4 h. Cells were then incubated with Fc Block for 20 minutes, fixed with fixation buffer (BD Bioscience) for 10 min at 37 °C and permeabilized with Perm/Wash buffer (BD Bioscience) for 20 min on ice. After that, cells were stained with anti-human granzyme B PE (clone GB11) (BD Bioscience) for an additional 30 minutes. For pSTAT3 staining, purified human CD8 memory T cells were incubated with RSV (5 × 10² PFU/ml) for 1 h. After that cells were stimulated with IL-21 (25 ng/ml) or LPS O111:B4 (50 ng/ml) for 30 min. Cells were then incubated with Fc Block for 20 minutes and stained with viability dye (Live/dead fixable violet dead cell stain – ThermoFisher Scientific) 1/10 for 15 minutes. Afterwards cells were stained with anti-human CD8 APC (clone RPA-T8) for 30 minutes, fixed with Cytofix (BD Bioscience) and subsequent permeabilization with Perm III BD Bioscience). For intracellular staining cells were incubated with anti-phospho-STAT3 (Ser727) polyclonal antibody (PA5-17876 – ThermoFisher Scientific) followed by secondary staining with goat anti-mouse IgG1 PE antibody (ThermoFisher Scientific). Samples were acquired on a FACS Canto II flow cytometer (BD Bioscience), and the data were analyzed using FlowJo version X.0.7 software (TreeStar).

### Cytotoxicity

Human pulmonary cells (MRC-5; ATCC CCL-171) (1 × 10^5^ cells) were cultured in 96-well plates for 24 h. After complete confluence, cells were infected with RSV (10^4^ PFU/ml) for 1 h. Afterwards, the medium was refilled, and human memory CD8 T cells (8 × 10^4^ cells) were seeded in coculture with infected MRC-5 cells and then stimulated with IL-21 (25 ng/ml) in RPMI with 2% FBS. To perform this assay, we added 3 conditions to the plate as indicated by the protocol for the LDH-Glo Cytotoxicity Assay (J2380 – Promega, WI, USA): maximum LDH release (untreated/uninfected MRC-5 cells); medium alone (wells containing only medium but no cells); and vehicle-only cells control (untreated/uninfected MRC-5 cells cocultured with memory CD8 T cells). Twelve hours after IL-21 addition, in the maximum LDH release wells, 10 μL of 10X lysis solution was added and allowed to incubate for 45 minutes at 37 °C in a 5% CO_2_ incubator. After that, the plate was centrifuged at 1500 rpm for 5 minutes, and 50 μL of culture supernatant from each well was carefully transferred into a new 96-well plate. Afterwards, 50 μl of the LDH detection reagent was added to the supernatants and then incubated at room temperature for 30 minutes protected from light. The plate was read at 520 nm in an ELISA reader (Biochrom). The percentage of cytotoxicity was calculated using the following formula: % Cytotoxicity = 100 × (Experimental LDH Release – Medium Background)/(Maximum LDH Release Control – Medium Background).

### Western blot analysis

Human pulmonary cells (MRC5; ATCC CCL-171) (1 × 10^5^ cells) were cultured in 96-well plates for 24 h. After complete confluence, cells were infected with RSV (10^4^ PFU/ml) for 1 h. Afterwards, human memory CD8 T cells (8 × 10^4^ cells) were placed in coculture with infected MRC-5 cells and then stimulated with IL-21 (25 ng/ml) for 30 min in RPMI 2% FBS. As a negative control, uninfected MRC-5 cells were used. Only the supernatant containing the memory CD8 T cells was carefully collected and centrifuged at 1000 rpm for 5 min. Total protein was extracted using a lysis buffer (10 mM Tris-HCl, pH 7.5; 1 mM MgCl_2_; 1 mM ethylenediaminetetraacetic acid [EDTA]; 0.1 mM phenylmethylsulfonyl fluoride [PMSF]; 5 mM 2-mercaptoethanol; 0.5% 3-[(3-cholamidopropyl)dimethylammonio]-1-propanesulfonate [CHAPS]; 10% glycerol). Briefly, samples were kept on ice for 40 minutes, vortexed every 10 minutes, and then centrifuged at 13000 rpm for 1 hour, and the supernatant containing the extracted protein was collected. Protein samples (15 μg) were separated on 10% SDS-PAGE polyacrylamide gels and transferred to nitrocellulose membranes (Bio-Rad). Later, the membrane was stained with 1:500 of phospho-STAT3 (Ser727) polyclonal antibody (ThermoFisher Scientific, MA, USA) or mouse anti-human β-actin monoclonal antibody (A2228 – Sigma Aldrich, Germany) followed by secondary staining with 1:1000 of rabbit anti-mouse IgG (H + L)-HRP antibody (ThermoFisher Scientific, MA, USA). Membrane blocking steps and antibody dilutions were performed using 5% (v/v) skim milk in 1X PBS, and washing steps were performed with Tween-20. Western blots were visualized by enhanced chemiluminescence (GE Healthcare Life Sciences). For the quantification of the bands, ImageJ software was used. β-Actin was used to normalize protein quantification, and the percentage was calculated over the untreated/uninfected control.

### In silico interaction analysis

The three-dimensional structures of RSV F protein (3RRR), RSV G protein (6BLH), and IL-21:IL-21R complex (3TGX) were obtained from Protein Data Bank (PDB). The RSV G protein structure available in PDB refers to a portion of 35 residues in length, which contains a central region of thirteen amino acids that are conserved across all RSV strains and directly involved in RSV attachment during infection^37–39^. The IL-21:IL21R was split into two files, each one containing one structure, using the UCSF Chimera software. The ClusPro 2.0 server^40–42^ was used to predict protein-protein interaction between IL-21R and the following proteins: IL-21 (positive control), F protein, and G protein. The results from ClusPro were downloaded and analyzed qualitatively (visual inspection of the interaction) and quantitatively (ClusPro model scores). The electrostatic potential computation and the generation of images were performed with both, UCSF Chimera and UCSF Chimera X^43,44^.

### Statistical analysis

All samples were first subjected to the Shapiro-Wilk normality test. A level of significance of *p* < 0.05 was established for the analyses. GraphPad Prism version 6.00 for Windows (GraphPad Software) was used for all statistical analyses and graphing.

## Supplementary information

Supplementary Material

## Data Availability

All data generated or analyzed during this study are included in this published article.
