# Supplementary material for: Respiratory syncytial virus reduces STAT3 phosphorylation in human memory CD8 T cells stimulated with IL-21
Source: Sci Rep. 2019 Nov 28;9:17766. doi: 10.1038/s41598-019-54240-9 (PMC6882881; doi:10.1038/s41598-019-54240-9)
Supplement: Supplementary file 1 — Supplementary Material [file 41598_2019_54240_MOESM1_ESM.pdf]

SUPPLEMENTARY MATERIAL

Respiratory syncytial virus reduces STAT3 phosphorylation in human memory CD8 T cells stimulated with IL-21

Krist Helen Antunes[1], André Becker[1], Caroline Franceschina[1], Deise do Nascimento de Freitas[1], Isadora Lape[1], Mariana D'Ávila da Cunha[1], Lidiane Leitão[2], Mauricio M. Rigo[1], Leonardo Araújo Pinto[2], Renato T. Stein[2], and Ana Paula Duarte de Souza[1,3]*.

1 Laboratory of Clinical and experimental Immunology, Infant Center, School of Medicine, Pontificia Universidade Católica do Rio Grande do Sul (PUCRS), Porto Alegre, Brazil.
2 Laboratory of Respiratory Physiology, Infant Center, School of Medicine, PUCRS
3 School of Health Sciences, PUCRS

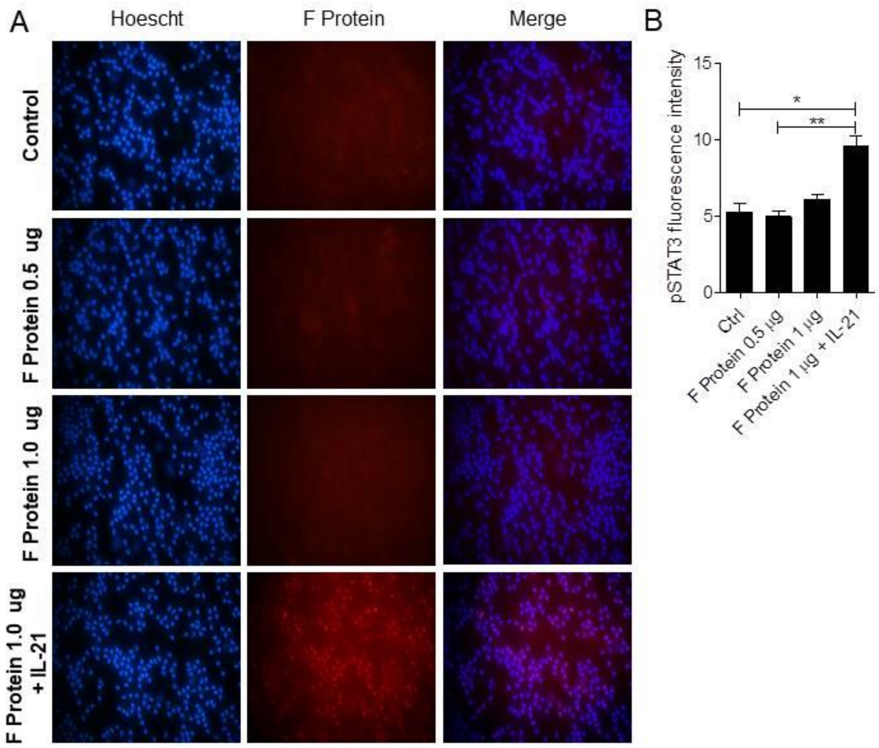

Supplementary Figure 1. RSV F protein does not inhibit pSTAT3 induced by IL-21 in purified human T CD8 cells. A-B, Human memory T CD8 cells were isolated from PBMCs, treated with RSV F protein (1 ug or 0.5 ug) for 1h and treated with IL-21 (25 ng/ml). After 30 min cells were harvest, fixed and stained for immunofluorescence assay. A, Fluorescence images of cell nuclei using hoescht (blue) and pSTAT3 Ser272 (red). B, Quantification of phosphorylated STAT3 in S2772 in purified human T CD8 cells. Data are expressed in mean ± SEM. Statistical significance was determinate using one-way ANOVA followed by Tukey's multiple comparison test. *$p < 0.05$, **$p < 0.01$.

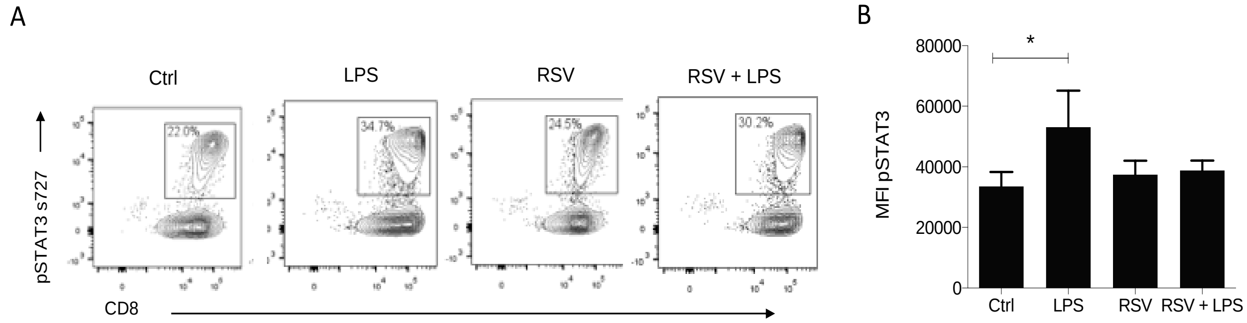

Supplementary Figure 2: RSV partially inhibits pSTAT3 induced by LPS in living purified human memory CD8 T cells. Human memory CD8 T cells were isolated from PBMCs, incubated with RSV (5x10² PFU/ml) for 1 h and treated with LPS O111:B4 (50 ng/ml). After 30 min, the cells were harvested and stained for flow cytometry analysis. A- Gate strategy and representative plots of flow cytometry analysis of pSTAT3 in memory CD8 T cells. B- MFI (mean of fluorescence intensity) of pSTAT3 in memory CD8 T cells. Data are expressed as the mean ± SEM. Statistical significance was determined using one-way ANOVA followed by Tukey's multiple comparison test. *$p < 0.05$.

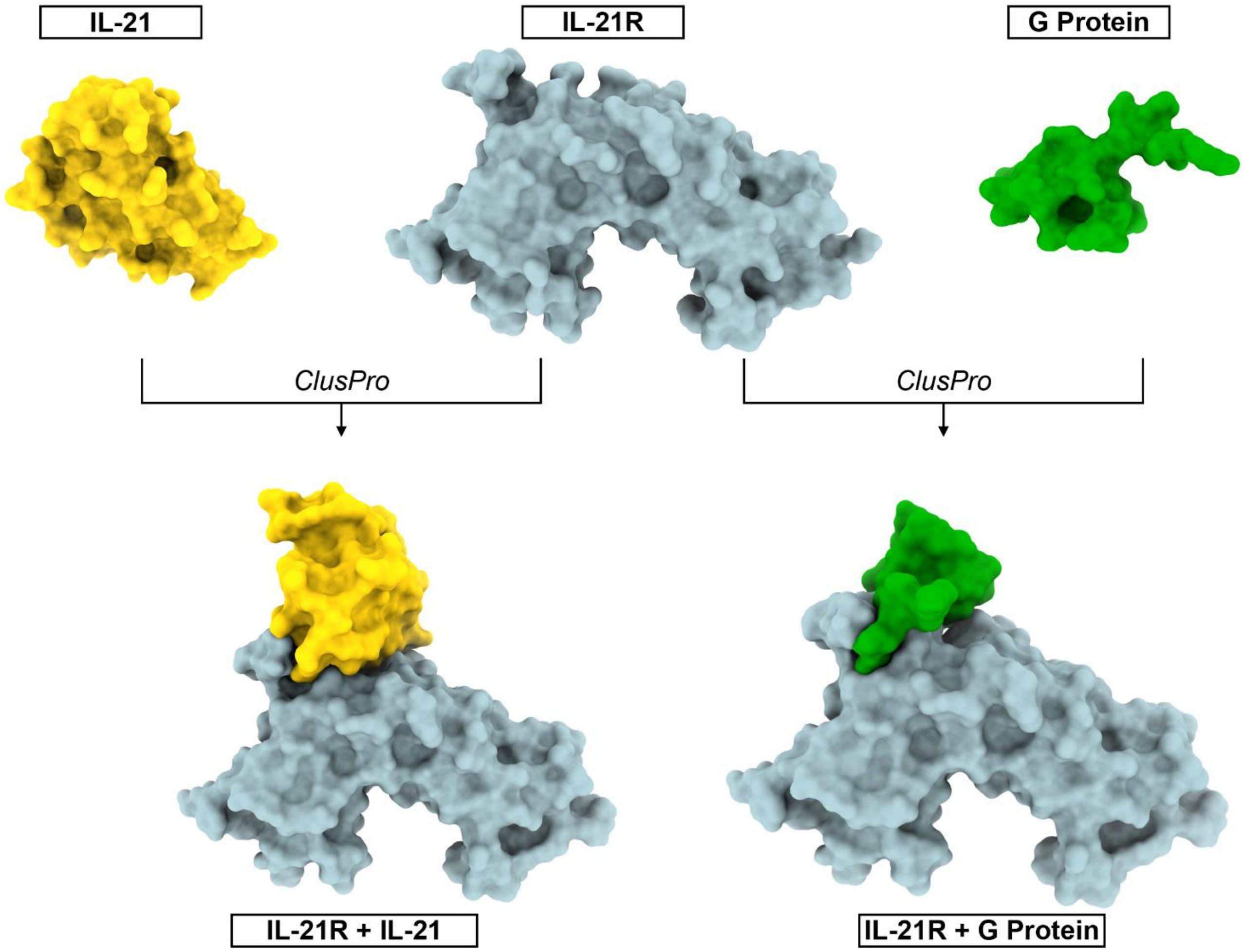

Supplementary Figure 3. IL-21 and RSV G protein share the same interaction site at IL-21R. The molecules were submitted to ClusPro server to simulate the possible binding modes of each complex.

A

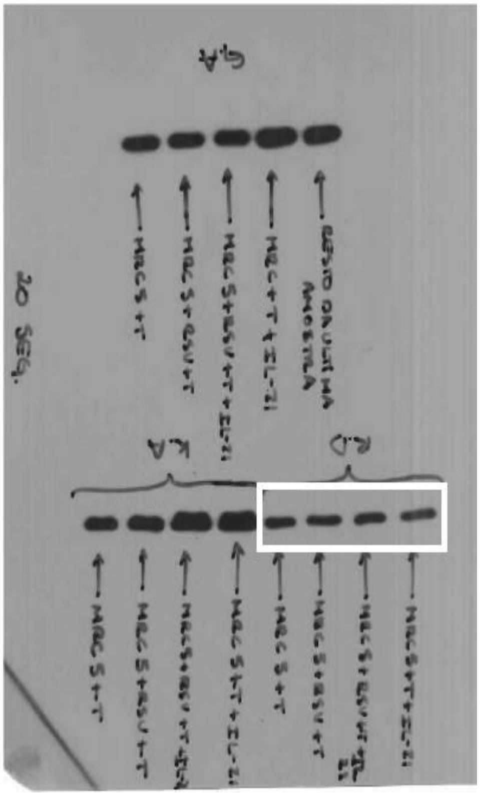

B

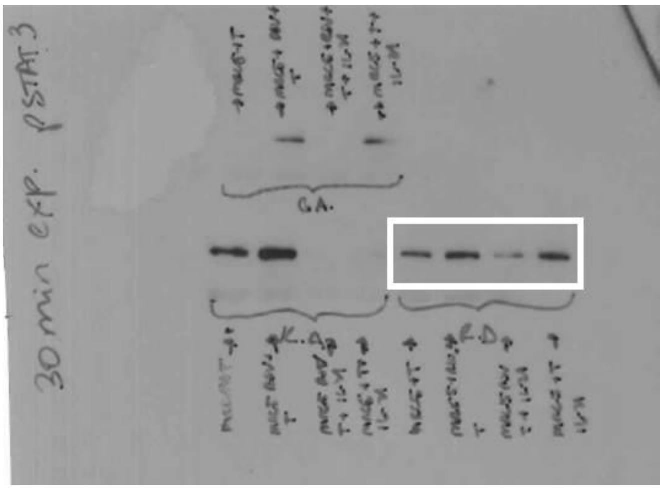

Supplementary Figure 4. Full-length lanes of Western blotting analysis. A, Full-length blot of b-actin detection (42 kD) and (B) pSTAT3 S727 detection (92 kD) from three different donors (R.D., K.A., and G.A.). The white squares indicate the cropped lanes showed in the Figure 3. The experiment was performed three times.
